# Case Report: Practical approach to differentiating juvenile parkinsonism of genetic cause and drug-induced parkinsonism in adolescents: a case series and literature review

**DOI:** 10.3389/fgene.2025.1672445

**Published:** 2025-10-10

**Authors:** Ioana Grigore, Lăcrămioara Ionela Butnariu, Thomas Gabriel Schreiner, Vasile Valeriu Lupu, Ancuta Lupu, Ludmila Darie, Cătălin Prăzaru, Elena Țarcă, Setalia Popa, Ecaterina Grigore

**Affiliations:** ^1^ Neurology, “St. Mary” Children Emergency Hospital, Iasi, Romania; ^2^ Department of Medical Genetics, Faculty of Medicine, Grigore T. Popa University of Medicine and Pharmacy, Iasi, Romania; ^3^ Department of Medical Specialties III, Faculty of Medicine, Grigore T. Popa University of Medicine and Pharmacy, Iasi, Romania; ^4^ Department of Pediatrics, Faculty of Medicine, Grigore T. Popa University of Medicine and Pharmacy, Iasi, Romania; ^5^ Department of Surgery II—Pediatric Surgery, Grigore T. Popa University of Medicine and Pharmacy, Iasi, Romania; ^6^ Faculty of Medicine, Grigore T. Popa University of Medicine and Pharmacy, Iasi, Romania

**Keywords:** juvenile parkinsonism, drug-induced parkinsonian syndrome, adolescents, genetic testing, extrapyramidal syndrome

## Abstract

Juvenile parkinsonism is a disease which is rarely seen in pediatric patients. In addition, drug-induced Parkinson’s syndrome is generally rare in children; however, it represents one of the most severe iatrogenic movement disorders in adults. The authors present one case of genetically confirmed juvenile parkinsonism and two cases of chronic Parkinson’s syndrome induced by psychiatric medication. The aim is to underline the differences between the clinical presentation, laboratory results, and the evolution under treatment. In addition, by reviewing literature data we analyzed the most significant information to describe the differences between these two conditions. Parkinsonian syndromes in pediatric patients and juvenile parkinsonism are difficult to diagnose based solely on clinical presentation because the manifestations are non-specific. Genetic testing and exclusion of other causes is essential for the final diagnosis. Adolescents who are diagnosed with drug-induced parkinsonian syndromes should undergo neurological evaluation, due to the fact that there is a risk of developing idiopathic Parkinson’s disease later during adulthood.

## 1 Introduction

Juvenile parkinsonism (JP) refers to patients whose symptoms begin to manifest before the age of 20 (Li et al., 2020; Niemann and Jankovic, 2019; Magistrelli et al., 2022; Riboldi et al., 2022). The average age on onset in JP is 10 years old, with a slightly higher prevalence in males compared to females (Morales-Briceño et al., 2020). Genetic testing have identified numerous mutations in relation to JP, such as mutations in the *SNCA* gene (located on chromosome 4q22.1) and *GCH1* gene (located on chromosome 14q22.2) with autosomal dominant (AD) inheritance, *PRKN (PARK2)* gene (located on chromosome 6q26), *TH* gene (located on chromosome 11p15.5) and *DJ1* gene (located on chromosome 1p36.32) with autosomal recessive (AR) inheritance, *PINK1* gene (located on chromosome 1p36.12) with either AD or AR inheritance (Morris and Lim, 2004).

Clinical manifestations typically include asymmetrical resting tremor, rigidity, dystonia, and gait disturbances. Depending on the genetic mutation, patients may also present with pyramidal signs, neuropathy, epileptic seizures, as well as intellectual disability (Morales-Briceño et al., 2020).

Drug-induced parkinsonian syndromes (DIP) represent the most common type of secondary parkinsonism and have initially been described as a complication of antipsychotic medication (Feldman et al., 2022). Subsequently, they have been reported as adverse effects of antiemetic drugs, anticholinergic medications, antidepressants, antiarrhythmic drugs, antivertigo products, and calcium channel antagonists (Kikegawa et al., 2024). Literature data support the fact that the onset of extrapyramidal signs during treatment with one of the abovementioned medications is one diagnostic criterion for DIP (Conn and Jankovic, 2024). In such cases, the symptoms should disappear within 6 months since discontinuation of medication, when this is possible (Chouksey and Pandey, 2020; Powell et al., 2020).

In both JP and DIP, other causes of extrapyramidal syndromes in pediatric patients have to be excluded, such as: cerebral vascular malformations, single lesions of basal nuclei, supratentorial tumors, metastatic cerebral lesions, obstructive hydrocephalus (den Heijer et al., 2020), hepatic problems (Wilson’s disease), systemic lupus erythematosus, and infectious diseases (Jain et al., 2021; Riboldi et al., 2022).

We present the cases of one girl and two boys who were evaluated neurologically due to the onset of hypertonic-hypokinetic extrapyramidal syndromes. Among them, the girl was the only one diagnosed with genetically confirmed JP, while the boys–with DIP.

We compared the clinical manifestations and the evolution under treatment of the three patients, highlighting the differences observed. To date, the diagnostic criteria for DIP have not been standardized and also the differences between JP and DIP are not yet presented in the literature.

## 2 Case presentation

### 2.1 Case 1

A female adolescent aged 14 years and 3 months underwent neurological examination because she accused symptoms which had been manifesting for approximately 1 year, with a slowly progressive evolution. These clinical symptoms consisted of postural instability, gait disorder, muscular rigidity and slowness in initiating and executing movements. The latter was initially affecting the left side, with more noticeable involvement of the lower left limb compared to the upper left limb, and later became bilateral, being associated with resting tremor in the limbs, which was accentuated by psychological stress. The patient also described states of anxiety, attention deficit, and memory impairment with insidious onset.

She was not following psychiatric treatment or any other chronic treatment. Family history was negative for neurological disorders.

Physical clinical examination revealed normal height and weight development, inexpressive facies, and slow blinking.

Neurological examination highlighted a hypertonic-hypokinetic extrapyramidal syndrome (bradykinesia, walking with small steps and a flexed posture, plastic hypertonia in all four limbs, cogwheel sign present bilaterally, being more accentuated on the left side compared to the right, exaggerated postural reflexes on the left side compared to the right, resting and postural tremor bilaterally, predominantly in the distal segments of the upper limbs, being accentuated by emotional states), symmetric deep tendon reflexes bilaterally, plantar reflex in flexion bilaterally, no cranial nerve lesions. The adolescent struggled to write, and the writing changed itself over time, the letters becoming smaller and smaller.

Common hematological and biochemical blood tests, ceruloplasmin, total copper, thyroxine (T4), thyroid-stimulating hormone (TSH) were in normal ranges. Inflammatory biological syndrome was absent.

Electroencephalogram (EEG) recorded a normal trail both spontaneously and during stimulation. Ophthalmologic examination was normal, with the absence of the Kayser-Fleischer ring.

Abdominal and thyroid ultrasounds did not reveal any pathological modifications.

**FIGURE 1:**
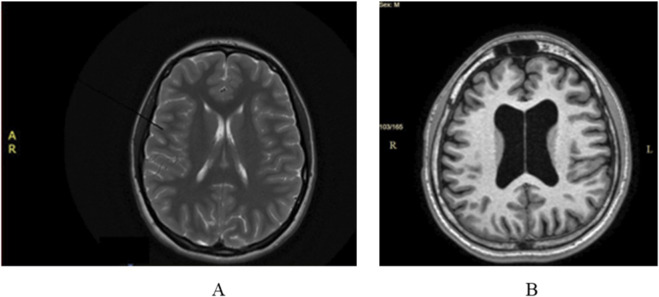
**(A)** Case 1 normal cerebral MRI; **(B)** Case 2. Cerebral MRI: ventriculomegaly and global cerebral atrophy.

Cerebral magnetic resonance imaging (MRI) did not show any pathological modifications apart from 6 mm of fluid accumulation at the medial wall of the right maxillary sinus (Figure 1A).

The initial diagnosis was parkinsonian syndrome with etiological investigations in progress–observation of JP. As imaging and laboratory tests excluded any other cause for the symptoms, genetic evaluation was recommended.

Genetic evaluation revealed normal height, weight, and head circumference, talus valgus, flat feet, kyphoscoliosis, and extrapyramidal syndrome. Whole exome sequencing (WES) (Blueprint Genetics Laboratory) was recommended and carried out. The result consisted of a homozygous pathogenic variant in the *PARK2* c.101_102del, p.(Gln34Argfs*5), which confirmed the diagnosis of JP, type 2 (OMIM 600116), with AR inheritance. Genetic testing of the parents was recommended, which revealed that both parents are healthy heterozygous carriers for the variant detected in the child.

Treatment was initiated with trihexyphenidyl hydrochloride (Romparkin^®^) 2 mg/tablet (initial dose of 1 tablet twice a day, which was increased after 4 months due to persistence of extrapyramidal symptoms to 2 and a half tablets daily). Patient’s evolution under treatment was favorable.

At the age of 16 years and 8 months, the patient reported worsening of bradykinesia and gait disorders, together with increased frequency of tremor episodes, without dyskinesia under the abovementioned treatment. Neurological examination was performed, and it was decided that Propranolol (20 mg/tablet) be added to the treatment plan, half a tablet twice a day, in the morning and either in the afternoon or at lunch. The patient was instructed to have the blood pressure and heart rate periodically evaluated by the general practitioner.

The evolution was favorable under treatment with Romparkin^®^ and Propranolol. At the age of 17 years and 7 months, neurological evaluation highlighted improvement of extrapyramidal symptoms and gait, together with a significant reduction of resting and postural tremor, and a better quality of life.

### 2.2 Case 2

A male adolescent aged 15 years and 3 months presented with resting tremor of the upper limbs with a recent onset. Family history was negative for neurological disorders.

From patient’s medical history it is worth mentioning one generalized epileptic seizure triggered by an episode of fever at the age of 1 year and 1 month. Additionally, the boy was registered in the Medical Genetics Department for Klinefelter syndrome and was under psychiatric observation for mild intellectual disability, attention deficit hyperactivity disorder (ADHD), and behavioral disorder, for which he received treatment with Aripiprazole 10 mg daily for approximately 2 months.

General physical examination revealed phenotypic features specific to Klinefelter syndrome associated with thoracolumbar scoliosis.

Neurological examination highlighted bradykinesia, plastic hypertonia of the limbs with cogwheel sign present bilaterally, postural and resting tremor of the upper limbs distally and bilaterally, which exacerbated during emotional stress but was absent during voluntary movement and sleep.

Laboratory investigations, including complete blood count, liver, kidney and thyroid function tests, glycemia, and lipid profile were within normal ranges. Inflammatory biological syndrome was absent.

Wilson’s disease was excluded based on the absence of the Kayser-Fleischer ring at ophtalmologic examination and normal plasma levels of ceruloplasmin and total copper. Abdominal and thyroid ultrasounds were normal.

Cerebral Computed tomography (CT) was normal, not showing any evidence of expansive brain processes, ischemic lesions, or hemorrhage.

Thoracolumbar spine MRI was suggestive for Scheuerman’s disease and showed circumferential protrusion of the vertebral disc L4-L5, which comes into minimal contact with the left root of L4 spinal nerve. It also revealed dextroconvex thoracolumbar scoliosis.

**FIGURE 2:**
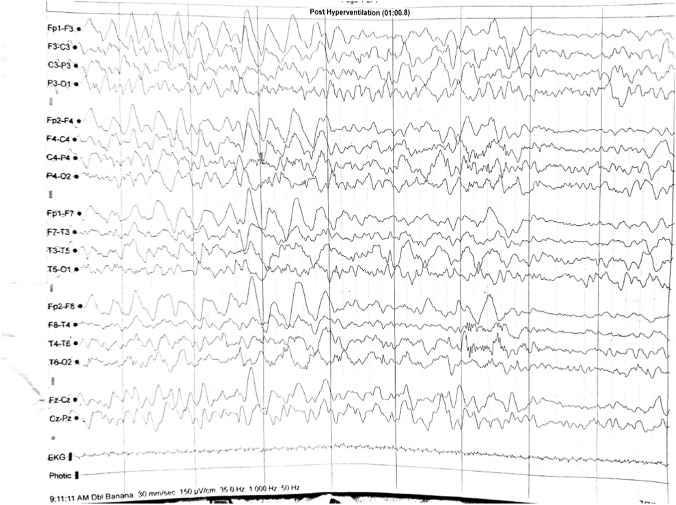
EEG (case 2): generalized high-voltage slow-waves during hyperventilation.

EEG revealed generalized high-voltage slow-waves during hyperventilation (Figure 2).

The results of clinical and laboratory investigations, which excluded an organic disease, and the chronic treatment with Aripiprazole led to the conclusion that the parkinsonian syndrome was secondary to antipsychotic drugs. Therefore, treatment with Romparkin^®^ (2 mg daily) was established and it was advisable to reduce or even discontinue the psychiatric medication. The former treatment was discontinued after a psychiatric consultation. After 6 months under treatment with Romparkin^®^, at the age of 15 years and 9 months, the patient had a complete remission of extrapyramidal symptoms. Romparkin^®^ treatment was discontinued.

The adolescent underwent another neurological assessment at the age of 16 years and 1 month because the extrapyramidal symptoms reappeared under psychiatric treatment with Quentiapine (50 mg daily) for his ADHD. Reintroduction of the treatment with Romparkin^®^ and reduction or cessation of the psychiatric medication was recommended after psychiatric evaluation. The dose of Quentiapine could only be reduced, and not completely discontinued, and the parkinsonian syndrome improved under treatment with Romparkin^®^. Due to the fact that extrapyramidal symptoms reappeared after administrating another psychiatric medication, it has been taken into consideration that the patient presents high risk of developing preclinical PD. For this reason the patient was asked to undergo periodic neurological examinations every 3 months. No signs of worsening symptoms have been noticed to this day.

### 2.3 Case 3

A 16-year-old male adolescent, with negative family history for neurological disorders and undergoing psychiatric treatment with Risperidone (1 mg daily, for approximately 6 months) for ADHD, addressed the neurologist for tremor predominantly in the distal segments of both upper limbs. General physical examination revealed well-proportioned somatic development.

Neurological examination highlighted hypertonic-hypokinetic extrapyramidal syndrome with bradykinesia, plastic hypertonia in all limbs, cogwheel sign present bilaterally, postural and resting tremor predominantly in the upper limbs distally and bilaterally, which worsened in stressful situations.

Hematological tests, liver and kidney function tests, glycemia, ceruloplasmin, total copper, blood iron, T4, and TSH plasma level were all within normal ranges. Inflammatory biological syndrome was also absent.

Awake EEG was normal.

No pathological aspects were found at the ophthalmologic examination or at the abdominal and thyroid ultrasounds.

Cerebral MRI showed ventriculomegaly without neurosurgical indication and global cerebral atrophy (Figure 1B). Those imaging modifications were not considered to be significant in relationship with the extrapyramidal manifestations.

All clinical and laboratory findings led to the exclusion of any organic diseases. Given the chronic treatment with Risperidone, it was concluded that the parkinsonian syndrome was caused by the antipsychotic medication. Treatment with Romparkin^®^ was initiated, and it was advisable to reduce or even discontinue the treatment with Risperidone. The hyperkinetic and behavioral disorders worsened as the daily dose of risperidone was reduced. Therefore, the patient had to go back to the initial dose of 1 mg per day. The following neurological evaluations revealed a decrease of the extrapyramidal symptoms under treatment with Romparkin^®^.

## 3 Discussion

Establishing the certain diagnosis for both Parkinson disease (PD) and DIP is of great importance for the therapeutic strategy (Benharroch, 2024; Ward and Citrome, 2018; Waninger et al., 2020).

### 3.1 Clinical aspects of JP and DIP

According to Shin and Chung (Shin and Chung, 2012), there are three main clinical diagnostic criteria used in DIP: the presence of parkinsonian syndrome, the absence of clinical symptoms prior to initiating the trigger-medication, and symptom onset during such treatment. Another significant diagnosis criterion is the disappearance of clinical signs after a variable time span since the cessation of the trigger-medication. However, not all cases can be distinguished only based on the patient’s clinical presentation (Hassin-Baer et al., 2001; Yomtoob et al., 2018; Tsang and Walker, 2023), because bradykinesia, rigidity and tremor in PD and DIP may overlap (Ward and Citrome, 2018; Jeong et al., 2021).

As hyposmia is a non-motor sign typical for PD which may occur years before the onset of the motor symptoms (Kwak et al., 2024), there are clinical studies that have statistically analyzed the presence of this sensory sign in adult patients with PD and in those with DIP. The former presented hyposmia in 75% of cases (Gjerde et al., 2018; Domellöf et al., 2017; Leonhardt et al., 2019). In addition, a decrease of olfaction has been reported in patients with DIP (Masala et al., 2018; Sasaki and Horie, 2020).

In the presented cases there were slight dissimilarities in the clinical presentations (Table 1). For instance, the female patient diagnosed with JP presented a wider range of extrapyramidal symptoms compared to the other adolescents who were diagnosed with DIP, and her clinical signs were more pronounced on the left side compared to the right. Our findings are in accordance with literature data. Anwar et al. (2019) described the case of a 16-year-old adolescent diagnosed with JP after the genetic tests revealed a heterozygote mutation in the *ATP13A2* gene, who presented resting tremor in the left upper limb and difficulty maintaining walking balance with swaying on the left side, among other typical sings (reduced facial expression with deceased frequency of blinking, vertical nystagmus, rigidity, and bradykinesia) (Anwar et al., 2019). A prospective study monitoring 30 patients with early-onset PD documented the fact that dystonia was present in a greater number of cases than resting tremor (Riboldi et al., 2022; Jha et al., 2017). Also, the tremor was the most disturbing symptom in our female patient.

**TABLE 1:** Comparison between our case with Juvenile Parkinsonism and the cases with Drug-induced parkinsonism.

Criteria	Case 1	Case 2	Case 3
*Neurological symptoms*	- bradykinesia - walking with small steps and a flexed posture - plastic hypertonia in all four limbs - cogwheel sign present asymmetric bilaterally - exaggerated postural reflexes on the left side compared to the right - resting and postural tremor bilaterally - anxiety - attention deficit - memory impairment	- bradykinesia - plastic hypertonia of the limbs - cogwheel sign present symmetric bilaterally - postural and resting tremor	- bradykinesia - plastic hypertonia in all limbs - cogwheel sign present symmetric bilaterally - postural and resting tremor
*Physical clinical examination*	inexpressive facies, slow blinking	phenotype features specific to Klinefelter syndrome	-
*Other diagnoses*	-	mild intellectual disability, ADHD	ADHD
*Psychiatric medication*	-	Aripiprazole Quentiapine	Risperidone
*MRI/CT*	normal	normal	Ventriculomegaly without neurosurgical indication and global cerebral atrophy
*Genetic testing*	homozygous mutation of gene *PARK2* c.101_102del.p. (Gin34Argfs^*^5)	-	-
*Outcome*	Favorable with treatment	Initially favorable with treatment and discontinuation of psychiatric medication The extrapyramidal symptoms reappeared under psychiatric treatment	Decrease of the extrapyramidal symptoms under treatment, but psychiatric treatment remained the same

In the cases of the two male patients diagnosed with DIP, the main complaints which determined their arrival at the neurologist doctor was accentuated tremor in the upper limbs, and their clinical signs were symmetrical. Moreover, none of the boys had presented any extrapyramidal symptoms before the initiation of psychiatric treatments–the clinical manifestations had an onset afterwards.

None of our patients presented any sort of olfactory dysfunction or olfactory nerve lesions.

### 3.2 The role of dopamine transporter imaging and electroencephalography in JP and DIP

There is a number of studies carried out on adult patients which try to establish other differential diagnostic criteria between PD and DIP, apart from the clinical presentation.

Some authors consider that dopamine transporter (DAT) imaging is an efficient method that is able to tell the difference between PD and DIP. In patients with DIP there has not been observed a dopamine deficit in comparison with those with Parkinson’s disease, whose DAT level in the striatum nucleus is significantly lower even in the early stages (Shin and Chung, 2012; Pa et al., 2021). This is because motor signs appear when 60%–80% of dopaminergic neurons are affected (Xiromerisiou et al., 2025). However, the use of DAT imaging is limited by its higher cost, risk of radiation and prolonged scan time after the radiotracer injection for positron emission tomography scan or single-photon emission computerized tomography (Seo et al., 2024a). Due to high investigation costs, these methods could not be used in our cases.

Some authors are of the opinion that EEG could be used in order to differentiate PD from DIP because of the correlation between slow EEG activity and cerebral dysfunction (Emmady et al., 2025). There are studies involving adult patients that have demonstrated that EEG pattern in patients with PD can be different from the control group consisting of healthy individuals and that EEG recordings may also be of great use in monitoring the evolution of the disease (Waninger et al., 2020; Bosch et al., 2022; Chaturvedi et al., 2017; Cozac et al., 2016; Geraedts et al., 2018; Lee et al., 2023). In adult patients with PD, resting awake EEG may highlight an increased number of theta waves and a decrease of alpha waves (Benharroch, 2024; Bosch et al., 2022; Han et al., 2013). It is widely agreed that slow EEG activity in patients suffering from PD is associated with cognitive decline (Benharroch, 2024; Bosch et al., 2022; Chaturvedi et al., 2017; Cozac et al., 2016; Geraedts et al., 2018; Kemp et al., 2024; Zawiślak-Fornagiel et al., 2023). According to Seo et al. this association does not fully explain the EEG modifications in patients with DIP (Seo et al., 2024a). They have compared EEG modifications in adults with PD and DIP divided in three groups. The first group consisted of 18 patients with DIP, the second consisted of 43 patients with PD and the third - 12 healthy individuals. The results showed that theta waves were most frequent in the group with DIP, followed by the group with PD and then the group with healthy individuals, while alpha waves had an opposite disposition between the three groups. The authors consider that EEG changes may be useful when interpreted in relationship to the different clinical aspects when it comes to making a differential diagnosis between PD and DIP (Seo et al., 2024a). The same authors also studied modifications of quantitative EEG (QEEG) in adult patients with PD and DIP. They reached the conclusion that a number of QEEG parameters, such as the frequency of background activity and the presence of theta waves, may be of great use in differentiating between the two conditions (Seo et al., 2024b).

In our first and third cases the EEG recordings were normal, but in the second case there were pathological modifications represented by generalized high-voltage slow-waves during hyperventilation. However, these were not considered to be specific to DIP.

### 3.3 Genetic testing for the diagnosis of JP

In the case of the adolescent with JP the final diagnosis was based on genetic testing. WES analysis carried out at Blueprint Genetics Laboratory identified a homozygote frameshift mutation in *PARK2* c.101_102del, p.(Gln34Argfs*5), which confirmed the clinical diagnosis.

There are 68 individuals heterozygous for this variant in gnom AD (The Genome Aggregation Database). This genetic variant generates a frameshift in exon 2 (of a total of 12 exons), which leads to the formation of a premature stop codon. Consequently, it is widely considered that this may lead to the loss of normal protein function, either by the production of an incomplete, shorter protein, or by the disintegration of mRNA caused by the nonsense mutation, also known as nonsense - mediated mRNA decay. Mutations which associate loss-of-function of *PARK2* gene represent a known causing mechanism for juvenile parkinsonism (The Human Gene Mutation Database) (HGMD). The *PARK2* c.101_102del, p.(Gln34Argfs*5) variant is reported in Clin Var database (Variation ID: 425403) and has been identified in many patients with PD (PMID: 25833766, 10072423, 19636047, 27206984) (Ambroziak et al., 2015; Abbas et al., 1999; Pankratz et al., 2009; Gautier et al., 2016; The Genome Aggregation Database, 2025).

### 3.4 Diagnosis of DIP

In the cases of our male patients, the diagnosis of DIP was based on the clinical presentation, especially on the disappearance of the extrapyramidal symptoms after 6 months since discontinuation of the antipsychotic medication in the case where this was possible. According to literature data, symptoms of DIP may disappear in a variable number time interval of weeks-months after cessation of the trigger-medication. However, extrapyramidal symptoms may persist or even worsen in 10%–15% of cases (Shin and Chung, 2012). The prognosis of these patients can be represented by the following: complete DIP recovery without clinical recurrence, persistence of a non-progressive or a progressive parkinsonian syndrome, disappearance of the clinical signs with future recurrence in the absence of the trigger-medication (Shin and Chung, 2012). Some other authors consider that only patients whose parkinsonian syndrome disappears without future symptoms recurrences may be diagnosed with “pure” DIP, while those whose parkinsonian syndrome persists, worsens or reappears may be categorized in a pre-clinical state of PD (Akdemir et al., 2021).

## 4 Conclusion

Parkinsonian syndrome in pediatric patients can be caused by a wide range of disorders. Although it is rare, JP should be taken into consideration in children who present movement disorders, while genetic testing is absolutely essential for the final diagnosis. A detailed patient history can provide important pieces of information, such as the use of neuroleptic medications for various psychiatric conditions, which help orient the diagnosis towards parkinsonian syndromes in children. A quick and precise diagnosis of JP or DIP is important for treatment, prognosis, and counseling for the patients and their families.

## Data Availability

The datasets presented in this article are not readily available because of ethical and privacy restrictions. Requests to access the datasets should be directed to the corresponding author.

## Abbreviations

PD: Parkinson’s disease; JP: Juvenile parkinsonism; DIP: Drug-induced parkinsonian syndromes; WES: Whole exome sequencing; DAT: Dopamine transporter.

## References

[B1] Abbas N. Lücking C. B. Ricard S. Dürr A. Bonifati V. De Michele G. (1999). A wide variety of mutations in the parkin gene are responsible for autosomal recessive parkinsonism in Europe. French parkinson's disease Genetics Study Group and the European Consortium on Genetic Susceptibility in Parkinson's Disease. Hum. Mol. Genet. 8 (4), 567–574. 10.1093/hmg/8.4.567 10072423

[B2] Akdemir Ü. Ö. Bora Tokçaer A. Atay L. Ö. (2021). Dopamine transporter SPECT imaging in Parkinson’s disease and parkinsonian disorders. Turk J. Med. Sci. 51 (2), 400–410. 10.3906/sag-2008-253 33237660 PMC8203173

[B3] Ambroziak W. Koziorowski D. Duszyc K. Górka-Skoczylas P. Potulska-Chromik A. Sławek J. (2015). Genomic instability in the PARK2 locus is associated with parkinson's disease. J. Appl. Genet. 56 (4), 451–461. 10.1007/s13353-015-0282-9 25833766 PMC4617850

[B4] Anwar A. Saleem S. Akhtar A. Ashraf S. Ahmed M. F. (2019). Juvenile parkinson disease. Cureus 1 (8), e5409. 10.7759/cureus.5409 31632863 PMC6795374

[B5] Benharroch D. (2024). Drug induced parkinsonism as it compareswith parkinson disease. Adv. Clin. Med. Res. 5 (2), 1–4. 10.52793/ACMR.2024.5(2)-76

[B6] Bosch T. J. Groth C. Singh A. (2022). Resting-state low-frequency cerebellar oscillations can be abnormal in Parkinson’s disease. Cerebellum 21 (6), 1139–1143. 10.1007/s12311-021-01343-7 34755280

[B7] Chaturvedi M. Hat F. Gschwandtner U. Bogaarts J. G. Meyer A. Fuhr P. (2017). Quantitative EEG (QEEG) measures differentiate Parkinson's disease (PD) patients from healthy Controls (HC). Front. Aging Neurosci. 9, 3. 10.3389/fnagi.2017.00003 28167911 PMC5253389

[B8] Chouksey A. Pandey S. (2020). Clinical spectrum of drug-induced movement disorders: a Study of 97 patients. *Tremor Other Hyperkinet Mov*. (N Y) 10, 48. 10.5334/tohm.554 33178486 PMC7597587

[B9] Conn H. Jankovic J. (2024). Drug-induced parkinsonism: diagnosis and treatment. Expert Opin. Drug Saf. 23 (12), 1503–1513. 10.1080/14740338.2024.2418950 39419777

[B10] Cozac V. V. Gschwandtner U. Hatz F. Hardmeier M. Rüegg S. Fuhr P. (2016). Quantitative EEG and cognitive decline in Parkinson’s disease. Park. Dis. 2016, 9060649. 10.1155/2016/9060649 27148466 PMC4842380

[B11] den Heijer J. M. Cullen V. C. Quadri M. Schmitz A. Hilt D. C. Lansbury P. (2020). A large-scale full GBA1 gene screening in Parkinson’s disease in the Netherlands. Mov. Disord. 35, 1667–1674. 10.1002/mds.28112 32618053 PMC7540512

[B12] Domellöf M. E. Lundin K. F. Edström M. Forsgren L. (2017). Olfactory dysfunction and dementia in newly diagnosed patients with Parkinson's disease. Park. RelatDisord 38, 41–47. 10.1016/j.parkreldis.2017.02.017 28242255

[B13] Emmady P. D. Asuncion R. M. D. Anilkumar A. C. (2025). EEG abnormal waveforms. Treasure Island (FL): StatPearls Publishing. Available online at: https://www.ncbi.nlm.nih.gov/books/NBK557655/(accessed on June 12, 2025). 32491587

[B14] Feldman M. Marmol S. Margolesky J. (2022). Updated perspectives on the management of drug-induced parkinsonism (DIP): insights from the Clinic. Ther. Clin. Risk Manag. 18, 1129–1142. 10.2147/TCRM.S360268 36573102 PMC9789682

[B15] Gautier C. A. Erpapazoglou Z. Mouton-Liger F. Muriel M. P. Cormier F. Bigou S. (2016). The endoplasmic reticulum-mitochondria interface is perturbed in PARK2 knockout mice and patients with PARK2 mutations. Hum. Mol. Genet. 25 (14), 2972–2984. 10.1093/hmg/ddw148 27206984

[B16] Geraedts V. J. Boon L. I. Marinus J. Gouw A. A. van Hilten J. J. Stam C. J. (2018). Clinical correlates of quantitative EEG in Parkinson disease: a systematic review. Neurology 91 (19), 871–883. 10.1212/WNL.0000000000006473 30291182

[B17] Gjerde K. V. Müller B. Skeie G. O. Assmus J. Alves G. Tysnes O. B. (2018). Hyposmia in a simple smell test is associated with accelerated cognitive decline in early Parkinson's disease. Acta Neurol. Scand. 138 (6), 508–514. 10.1111/ane.13003 30058142

[B18] Han C. X. Wang J. Yi G. S. Che Y. Q. (2013). Investigation of EEG abnormalities in the early stage of Parkinson's disease. CognNeurodyn 7 (4), 351–359. 10.1007/s11571-013-9247-z 24427211 PMC3713203

[B19] Hassin-Baer S. Sirota P. Korczyn A. D. Treves T. A. Epstein B. Shabtai H. (2001). Clinical characteristics of neuroleptic-induced parkinsonism. J. Neural Transm. 108, 1299–1308. 10.1007/s007020100006 11768628

[B20] Jain R. Pandey S. Raghav S. (2021). Movement disorders in children. Indian Pediatr. 58 (9), 861–870. 10.1007/s13312-021-2310-7 34016797

[B21] Jeong S. Cho H. Kim Y. J. Ma H. I. Jang S. (2021). Drug-induced parkinsonism: a strong predictor of idiopathic Parkinson's disease. PLoS One 16 (3), e0247354. 10.1371/journal.pone.0247354 33647030 PMC7920346

[B22] Jha V. N. Roy S. Singh P. K. (2017). Juvenile parkinsonism – a diagnostic dilemma. J. Neurol. Exp. Neurosci. 3 (1), 33–35. 10.17756/jnen.2017-025

[B23] Kemp A. F. Kinnerup M. Johnsen B. Jakobsen S. Nahimi A. Gjedde A. (2024). EEG frequency correlates with α_2_-Receptor density in Parkinson's Disease. Biomolecules 14 (2), 209. 10.3390/biom14020209 38397446 PMC10886955

[B24] Kikegawa M. Sone H. Uesawa Y. (2024). Comprehensive analysis of drug-induced parkinson-like events. Pharmaceuticals 17, 1099. 10.3390/ph17081099 39204204 PMC11359003

[B25] Kwak I. H. Kim Y. E. Kang S. Y. Lee J. S. Lee J. Kim M. S. (2024). Comparative olfactory profiles in Parkinson's Disease and drug-induced parkinsonism. J. Mov. Disord. 17 (1), 64–70. 10.14802/jmd.23105 37798852 PMC10846967

[B26] Lee H. Jeon Y. Yoo C. Seon H. Park J. Hwang M. (2023). Persistent impacts of smoking on resting-state EEG in male chronic smokers and past-smokers with 20 years of abstinence. Sci. Rep. 13 (1), 3907. 10.1038/s41598-023-29547-3 36890138 PMC9995515

[B27] Leonhardt B. Tahmasebi R. Jagsch R. Pirker W. Lehrner J. (2019). Awareness of olfactory dysfunction in Parkinson's disease. Neuropsychology 33 (5), 633–641. 10.1037/neu0000544 30945913

[B28] Li D. Aung-Htut M. T. Ham K. A. Fletcher S. Wilton S. D. (2020). A splice intervention therapy for autosomal recessive juvenile Parkinson's Disease arising from parkin mutations. Int. J. Mol. Sci. 21 (19), 7282. 10.3390/ijms21197282 33019779 PMC7582384

[B29] Magistrelli L. Contaldi E. Milner A. V. Gallo S. Sacchetti M. Fornaro R. (2022). A very early onset of juvenile parkinsonism. J. Neurol. 269 (12), 6661–6663. 10.1007/s00415-022-11278-6 35851923

[B30] Masala C. Solla P. Liscia A. Defazio G. Saba L. Cannas A. (2018). Correlation among olfactory function, motors' symptoms, cognitive impairment, apathy, and fatigue in patients with Parkinson's disease. J. Neurol. 265 (8), 1764–1771. 10.1007/s00415-018-8913-9 29804147

[B31] Morales-Briceño H. Mohammad S. S. Post B. Fois A. F. Dale R. C. Tchan M. (2020). Clinical and neuroimaging phenotypes of genetic parkinsonism from infancy to adolescence. Brain 143 (3), 751–770. 10.1093/brain/awz345 31800013

[B32] Morris H. Lim S. Y. (2004). “Monogenic parkinson disease overview,” in GeneReviews® Adam M. P. Feldman J. Mirzaa G. M. (Seattle (WA): University of Washington, Seattle), 1993–2025. Available online at: https://www.ncbi.nlm.nih.gov/books/NBK1223/(accessed on June 8, 2025). 20301402

[B33] Niemann N. Jankovic J. (2019). Juvenile parkinsonism: differential diagnosis, genetics, and treatment. Park. RelatDisord 67, 74–89. 10.1016/j.parkreldis.2019.06.025 31272925

[B34] Palermo G. Giannoni S. Bellini G. Siciliano G. Ceravolo R. (2021). Dopamine transporter imaging, Current status of a potential biomarker: a comprehensive review. Int. J. Mol. Sci. 22, 11234. 10.3390/ijms222011234 34681899 PMC8538800

[B35] Pankratz N. Kissell D. K. Pauciulo M. W. Halter C. A. Rudolph A. Pfeiffer R. F. (2009). Parkin dosage mutations have greater pathogenicity in familial PD than simple sequence mutations. Neurology 73 (4), 279–286. 10.1212/WNL.0b013e3181af7a33 19636047 PMC2715211

[B36] Powell A. Gallur L. Koopowitz L. Hayes M. W. (2020). Parkinsonism in the psychiatric setting: an update on clinical differentiation and management. BMJ Neurol. Open 2 (1), e000034. 10.1136/bmjno-2019-000034 33681781 PMC7871718

[B37] Riboldi G. M. Frattini E. Monfrini E. Frucht S. J. Di Fonzo A. (2022). A practical approach to early-onset parkinsonism. J. Park. Dis. 12 (1), 1–26. 10.3233/JPD-212815 34569973 PMC8842790

[B38] Sasaki S. Horie Y. (2020). Association between olfactory impairment and disease severity and duration in Parkinson's Disease. Mov. Disord. Clin. Pract. 7 (7), 820–826. 10.1002/mdc3.13028 33043078 PMC7534008

[B39] Seo S. Kim S. Kim S. P. Kim J. Kang S. Y. Chung D. (2024a). Low-frequency EEG power and coherence differ between drug-induced parkinsonism and Parkinson's disease. Clin. Neurophysiol. 168, 131–138. 10.1016/j.clinph.2024.10.013 39509953

[B40] Seo S. Kim S. Kim S. P. Kim J. Chung D. Kang S. Y. (2024b). Quantitative EEG analysis for comparing drug-induced parkinsonism and parkinson’s disease (abstract). Mov. Disord. 39 (Suppl. 1). Available online at: https://mdabstracts.org/abstract/quantitative-eeg-analysis-for-comparing-drug-induced-parkinsonism-and-parkindons-disease/(Accessed September 30, 2025).

[B41] Shin H. W. Chung S. J. (2012). Drug-induced parkinsonism. J. Clin. Neurol. 8 (1), 15–21. 10.3988/jcn.2012.8.1.15 22523509 PMC3325428

[B42] The Genome Aggregation Database (2025). The genome aggregation database. Available online at: https://gnomad.broadinstitute.org/(Accessed on June 8, 2025).

[B43] Tsang K. Walker R. (2023). Dopamine transporter single photon emission computed tomography (DaT-SPECT) use in the diagnosis and clinical management of parkinsonism: an 8-year retrospective study. J. Neurol. 270 (5), 2550–2558. 10.1007/s00415-023-11563-y 36795149 PMC10129961

[B44] Waninger S. Berka C. Stevanovic Karic M. Korszen S. Mozley P. D. Henchcliffe C. (2020). Neurophysiological biomarkers of parkinson’s disease. J. Parkinson’s Dis. 10 (2), 471–480. 10.3233/JPD-191844 32116262 PMC7242849

[B45] Ward K. M. Citrome L. (2018). Antipsychotic-Related movement disorders: Drug-Induced parkinsonism vs. tardive Dyskinesia—Key differences in pathophysiology and clinical management. Neurol. Ther. 7, 233–248. 10.1007/s40120-018-0105-0 30027457 PMC6283785

[B46] Xiromerisiou G. Boura I. Barmpounaki E. Georgoulias P. Dardiotis E. Spanaki C. (2025). The utilization and impact of dopamine transporter imaging in diagnosing movement disorders at a tertiary care Hospital in Greece. Biomedicines 13, 970. 10.3390/biomedicines13040970 40299648 PMC12024717

[B47] Yomtoob J. Koloms K. Bega D. (2018). DAT-SPECT imaging in cases of drug-induced parkinsonism in a specialty movement disorders practice. Park. RelatDisord 53, 37–41. 10.1016/j.parkreldis.2018.04.037 29748111

[B48] Zawiślak-Fornagiel K. Ledwoń D. Bugdol M. Romaniszyn-Kania P. Małecki A. Gorzkowska A. (2023). The increase of theta power and decrease of alpha/Theta ratio as a manifestation of cognitive impairment in parkinson’s disease. J. Clin. Med. 12, 1569. 10.3390/jcm12041569 36836103 PMC9965386

